# Spontaneous idiopathic omental haemorrhage: a rare cause of right iliac fossa pain

**DOI:** 10.1186/s40792-016-0163-4

**Published:** 2016-04-14

**Authors:** Nima Ahmadi, Jonathan S. Y. Hong, William S. Mackie

**Affiliations:** Department of Surgery, Orange Health Service, Orange, NSW Australia; Surgical Outcome Research Centre (SOuRCe), Royal Prince Alfred Hospital, Missenden Rd, Camperdown, 2050 Sydney, NSW Australia

**Keywords:** Omental haemorrhage, Haemoperitoneum

## Abstract

**Background:**

Isolated omental haemorrhage is a rare entity of which only case reports exist. This is usually in the setting of trauma, neoplasms or anticoagulation.

**Case presentation:**

We report a case of spontaneous idiopathic omental haemorrhage with no evidence of trauma, neoplasm or presence of anticoagulation. This was identified on the imaging studies performed for the purpose of diagnosis of the cause of the patient’s right iliac fossa pain. The patient required urgent laparotomy and omentectomy to achieve haemostasis.

**Discussion:**

Spontaneous omental haemorrhage is a rare entity that is usually preceeded by trauma or occurs in the context of adhesions, neoplasms or anticoagulation. If there are delays in diagnosis, it could lead to significant morbidity for the patient. Therefore, it requires prompt recognition and definitive management.

**Conclusion:**

Spontaneous omental haemorrhage is a rare entity characterised only in case reports. It is usually a secondary event and requires prompt management.

## Background

Omental haemorrhage leading to significant haemoperitoneum is a rare entity. Within the literature, the only publications of spontaneous omental bleeding are case reports [1–4]. The secondary causes include trauma, neoplasms [5], varices [6, 7], adhesions [8, 9], torsion [10], arterial aneurysm [11, 12], vasculitis [13] and omental pregnancies [14–16].

Idiopathic omental haemorrhage typically presents with severe sudden onset pain and occasional nausea, vomiting or diarrhoea [1–3]. This can mimic other more common causes of abdominal pain. The management involves initial resuscitation, correction of coagulopathy, if present, followed by imaging if the haemodynamics of the patient allow.

Imaging may include focused assessment with sonography, which has been used to detect acute intraperitoneal haemorrhage [17]. Contrast computed tomography is useful to localise the site of bleeding and exclude more common pathology [2, 3, 17]. Formal arterial angiography may permit ultra-selective embolisation, but its use is limited by availability of this modality [3]. However, within the literature, the majority of reported cases have proceeded to laparotomy and partial omentectomy.

Below, we report a case of spontaneous idiopathic omental rupture leading to haemoperitoneum needing operative management.

## Case presentation

A 53-year-old gentleman presented to our emergency department with a 4-h history of right iliac fossa and right periumbilical pain. There was no history of trauma. The onset of pain was sudden, and it was intermittent in nature and worse on movement. He denied nausea or other gastrointestinal symptoms.

The patient had background of atrial fibrillation (AF) but was not anticoagulated. There was no history of abdominal surgery.

On presentation, his heart rate was 100 bpm and he was in AF, with a blood pressure of 142/90 mmHg. He had focal peritonism in the right lumbar region and right hypochondrium.

Initial blood tests showed his haemoglobin to be 143 g/L, white cell count of 9.6 × 10^9^/L, platelet count of 270 × 10^9^ /L.

**Fig. 1:**
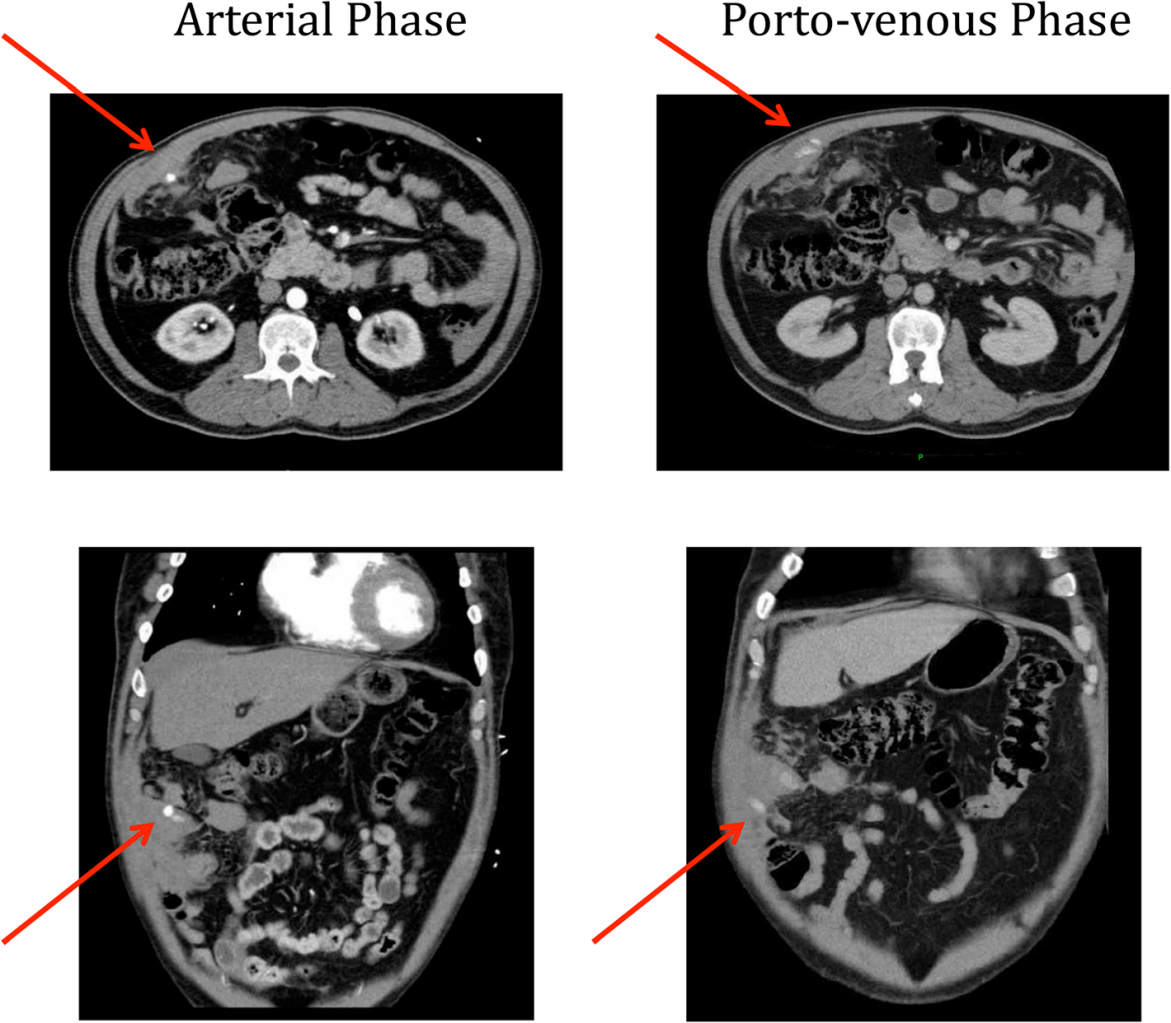
CT abdomen/pelvis with IV contrast with arterial and porto-venous phases demonstrating blush (*arrows*) and free high attenuation fluid in the peritoneum

A CT abdomen demonstrated free high attenuation fluid around the liver and the right paracolic gutter. An area of high attenuation was noted at the right lateral anterior abdominal wall with contrast extravasation on both arterial and porto-venous phases suspicious for an omental haemorrhage and haematoma (see Fig. 1).

At laparotomy, there was 2 L of free blood. There were no adhesions or varices. A large haematoma was noted within the right upper quadrant omentum, but there was no active bleeding. Within this segment of omentum, an abnormal area was identified as a possible point of bleeding. This was resected.

The patient was discharged after 4 days.

**Fig. 2:**
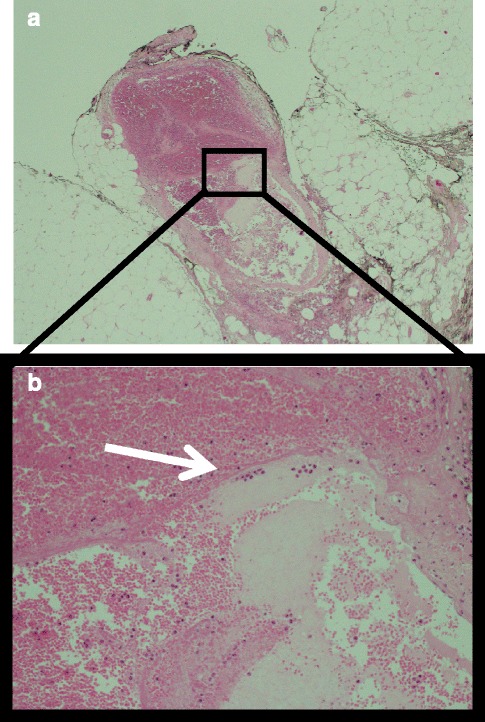
Histological slide with H&E staining (**a**). The magnified image (**b**) demonstrating a ruptured vessel (*arrow*) with extraluminal blood within the omentum. There was no evidence of neoplastic or vasculitic change

The histopathological demonstrated ruptured medium-sized vessels at the site of macroscopic abnormality, there was no vasculitis or malignancy (Fig. 2). The remainder of the specimen was histopathologically normal.

## Conclusion

Isolated, primary omental haemorrhage is a rare entity that is characterised only in case reports. Secondary causes include trauma, neoplasm, adhesion or anticoagulation.

Patients typically present with severe sudden onset pain and occasional nausea, vomiting or diarrhoea [1–3]. Management involves resuscitation, correction of coagulopathy, followed by imaging, if the patient is stable.

Imaging may include focused assessment with sonography, which has been used to detect acute intraperitoneal haemorrhage [17]. Contrast computed tomography is useful to localise the site of bleeding and exclude more common pathology [2, 3, 17]. Formal arterial angiography may permit ultra-selective embolisation, but its use is limited by availability of this modality [3].

The majority of reported cases proceed to laparotomy and partial omentectomy. There is one case report of a laparoscopic approach to management [18]; however, similar to our case, the surgeons had difficulty identifying the bleeding point and had to increase their incision to perform an extracorporeal evaluation of the omentum and perform a partial omentectomy. Similarly, in our case, there was no active bleeding identified at the time of the operation and our approach with a laparotomy and partial omentectomy permitted histopathological assessment of the affected omentum to exclude other causes of omental bleeding.

Given that spontaneous omental haemorrhage is such a rare entity, a stepwise approach should be taken for patients presenting with abdominal pain and it is wise to remember that common things occur commonly. However, one should always have an index of suspicion for identifying unexpected pathology as such in this case.

## Consent

Written informed consent was obtained from the patient for publication of this case report and any accompanying images. A copy of the written consent is available for review by the Editor-in-Chief of this journal.

## Abbreviations

AF: atrial fibrillation
CT: computed tomography
